# Supplementary material for: A rapid and sensitive assay for quantifying the activity of both aerobic and anaerobic ribonucleotide reductases acting upon any or all substrates
Source: PLoS One. 2022 Jun 8;17(6):e0269572. doi: 10.1371/journal.pone.0269572 (PMC9176816; doi:10.1371/journal.pone.0269572)
Supplement: S1 Table — Each temperature was assayed five times using 40 μL of water in a thin-walled PCR tube that had an initial temperature of 37°C. (DOCX) [file pone.0269572.s001.docx]

**S1 Table. Time taken for water to reach various temperatures upon insertion into a 95 °C thermocycler.** Each temperature was assayed five times using 40 µL of water in a thin-walled PCR tube that had an initial temperature of 37 °C.

| Temperature (°C)^a, b^ | Average Time to Temperature (s) | Standard Deviation Time to Temperature (s) |
| --- | --- | --- |
| 45 | 2 | 0 |
| 60 | 5 | 1 |
| 85 | 17 | 2 |

1. Temperature was determined by insertion of a thin probe attached to a multimeter set to the temperature setting.
2. The multimeter took 4 ± 1 seconds to register 85 °C when inserted into a solution already at 95 °C and <1 second to register 37 °C when inserted into a solution already at 37 °C. Temperatures higher than 85 °C could not be reliably measured by the multimeter and so were not included in this table. Since the melting temperature of the thermophilic protein of interest was approximately 50 °C (S1 Fig), the time to 95 °C was not pursued further since we were able to generate two data points to determine how long it took to reach a relevant temperature for the proteins at hand.
