# Supplementary material for: A rapid and sensitive assay for quantifying the activity of both aerobic and anaerobic ribonucleotide reductases acting upon any or all substrates
Source: PLoS One. 2022 Jun 8;17(6):e0269572. doi: 10.1371/journal.pone.0269572 (PMC9176816; doi:10.1371/journal.pone.0269572)
Supplement: S2 Table — (DOCX) [file pone.0269572.s002.docx]

S2 Table. MRM parameters for each of the four deoxyribonucleoside products and their corresponding internal standards quantified in the RNR reaction.

| Analyte | Precursor ion (m/z) | Product ion (m/z) | Fragmentor voltage (V) | Collision energy (V) | Approx. Retention Time (min) |
| --- | --- | --- | --- | --- | --- |
| dA | 252.1 | 135.9 | 80 | 20 | 5.0 – 5.7 |
| dC | 228.2 | 112.1 | 165 | 20 | 1.3 |
| dG | 268.1 | 152.3 | 80 | 20 | 1.4 – 1.6 |
| dU | 229.1 | 112.9 | 60 | 6 | 1.3 |
| ^13^C, ^15^N dA | 267.3 | 145.9 | 80 | 20 | 5.0 – 5.7 |
| ^13^C, ^15^N dC | 240.2 | 119.1 | 165 | 20 | 1.3 |
| ^13^C, ^15^N dG | 283.1 | 162.3 | 80 | 20 | 1.4 – 1.6 |
| ^13^C, ^15^N dU | 256.1 | 118.9 | 60 | 6 | 1.3 |
