# Supplementary material for: A rapid and sensitive assay for quantifying the activity of both aerobic and anaerobic ribonucleotide reductases acting upon any or all substrates
Source: PLoS One. 2022 Jun 8;17(6):e0269572. doi: 10.1371/journal.pone.0269572 (PMC9176816; doi:10.1371/journal.pone.0269572)
Supplement: S3 Table — (DOCX) [file pone.0269572.s003.docx]

S3 Table. LC-MS program used for the LC-MS/MS experiments.

| Time (min) | %B | Flow (mL/min) | Max pressure (bar) |
| --- | --- | --- | --- |
| 0 | 3 | 0.350 | 400 |
| 5 | 5 | 0.350 | 400 |
| 6 | 95 | 0.350 | 400 |
| 7 | 3 | 0.350 | 400 |
| 10 | 3 | 0.350 | 400 |
