# Supplementary figures and images for: A rapid and sensitive assay for quantifying the activity of both aerobic and anaerobic ribonucleotide reductases acting upon any or all substrates
Source: PLoS One. 2022 Jun 8;17(6):e0269572. doi: 10.1371/journal.pone.0269572 (PMC9176816; doi:10.1371/journal.pone.0269572)

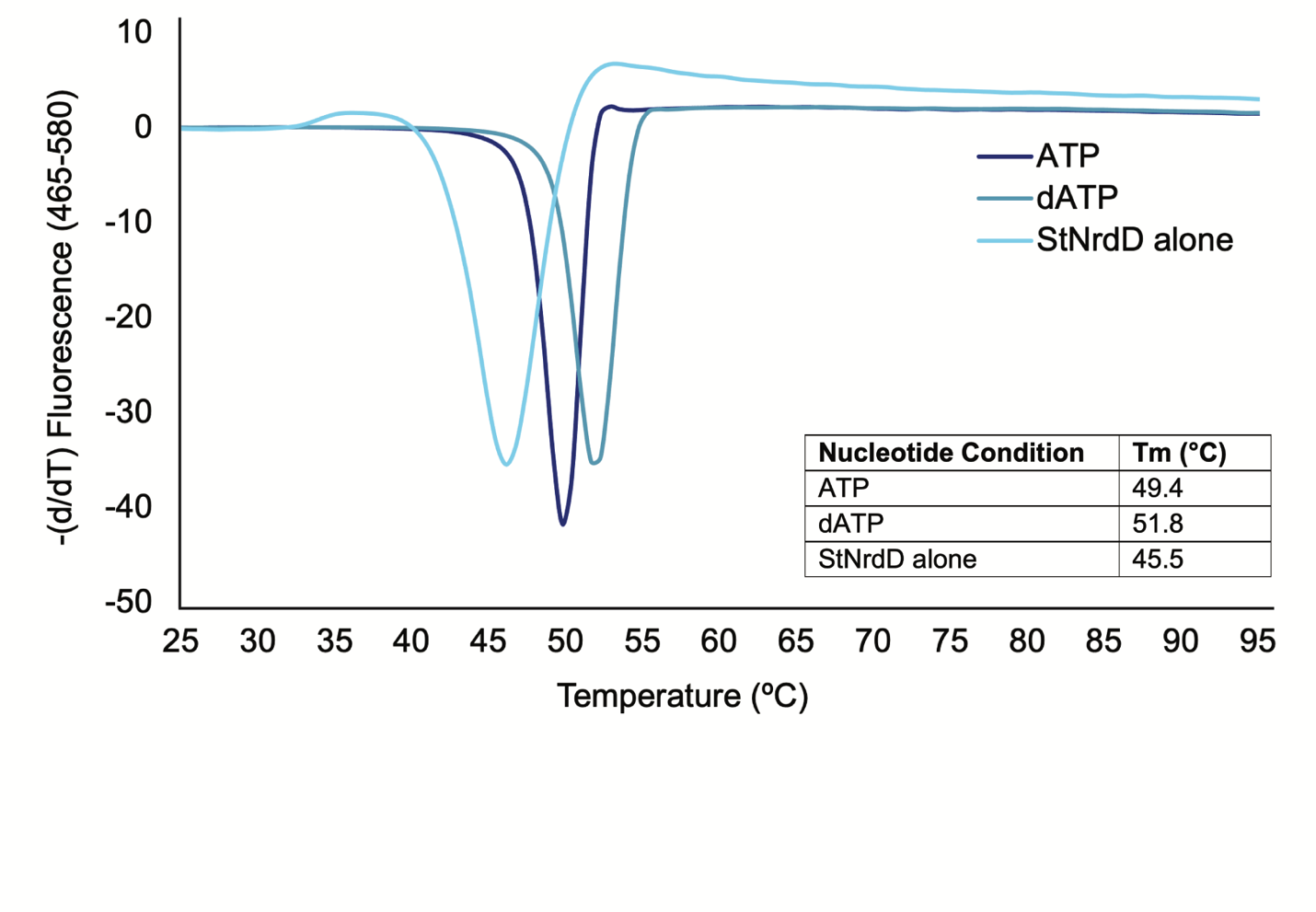

Supplement: S1 Fig — Protein melting peaks of StNrdD with no nucleotides (StNrdD alone) or 1 mM TTP and GTP and either 3 mM ATP or 3 mM dATP. Table insert summarizes the melting temperature of StNrdD under the three conditions, which equates to the temperature at the negative peak of each curve. (TIF) [file pone.0269572.s004.tif]

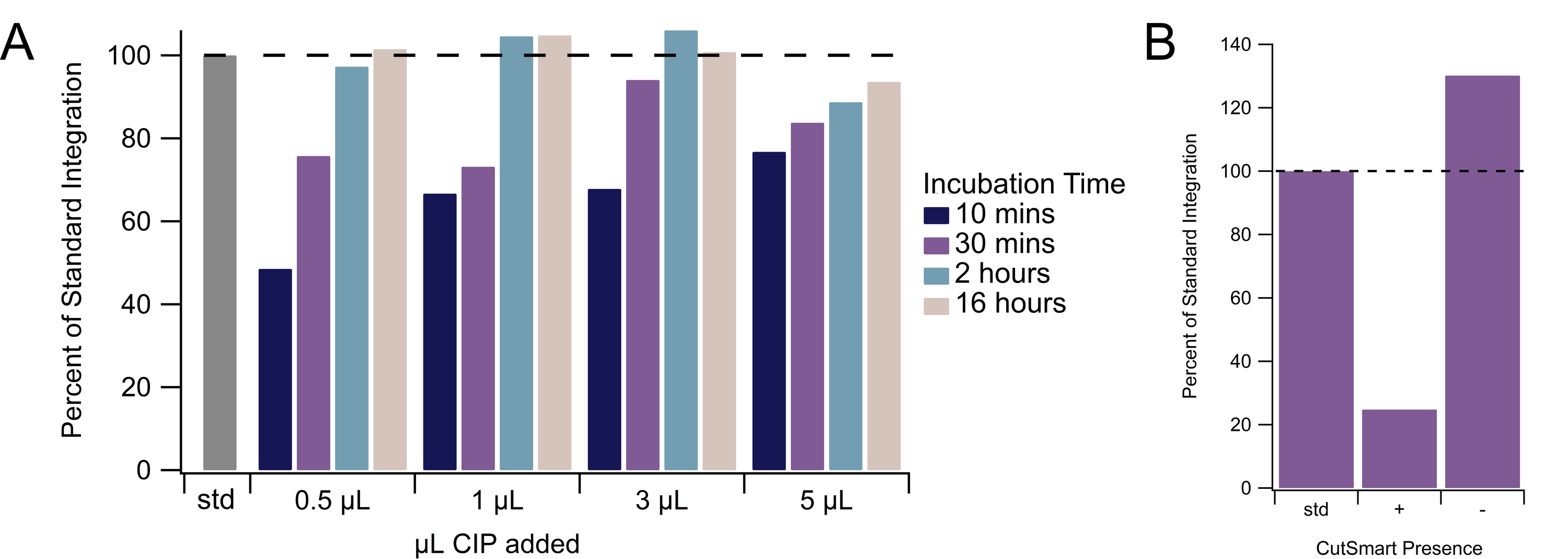

Supplement: S2 Fig — (A) Variation in dephosphorylation based on volume CIP added and incubation time (at 37°C) on a solution of 3 mM ATP and 1 mM CTP. Dephosphorylation was determined by adenosine MRM signal and was compared to a standard of 3 mM adenosine nucleoside. 1 μL CIP addition and incubation for 2 hours was used for all further studies. (B) Impact of the addition of CutSmart buffer to the CIP incubation mixture on a solution of 3 mM ATP and 1 mM CTP. Dephosphorylation was determined by cytidine MRM signal and was compared to a standard of 1 mM cytidine nucleoside. No CutSmart buffer was added in any further studies. (TIF) [file pone.0269572.s005.tif]

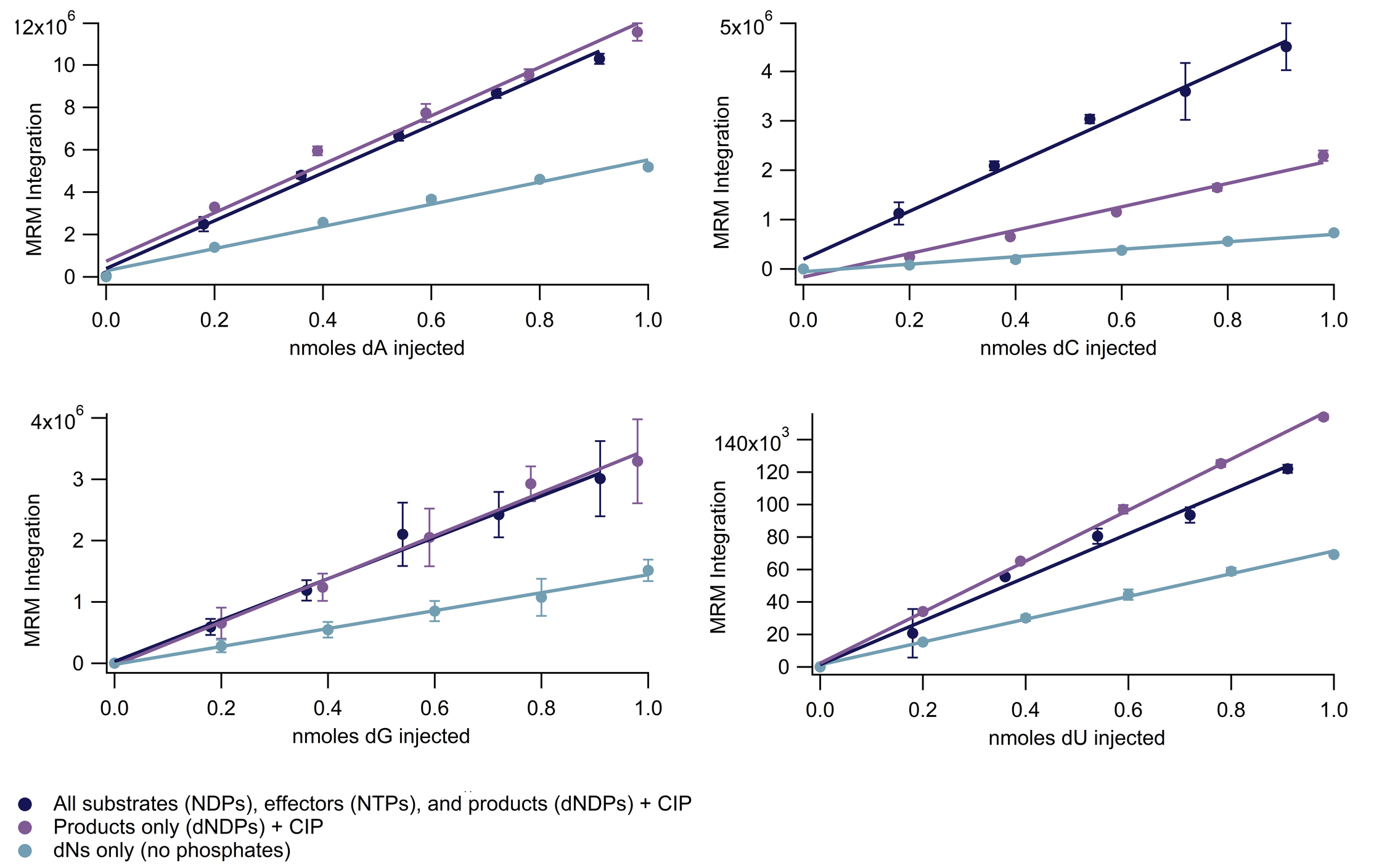

Supplement: S3 Fig — The presence of substrate and effector nucleosides and/or phosphates in addition to the measured deoxyribonucleoside standard is essential in generation of accurate standard curves. A comparison of dA, dC, dG, and dU standard curves generated either from deoxyribonucleotide triphosphates in the presence of substrates and effectors (navy), deoxyribonucleotide triphosphates but no substrates or effectors (purple), or only deoxyribonucleosides (no phosphates; light blue). For conditions where nucleotides were present, nucleotides were treated with CIP prior to analysis by mass spectrometry. Concentrations of substrate and effector nucleotides were as follows: ADP (110 μM), GDP (200 μM), UDP (50 μM), CDP (70 μM), ATP (3 mM). All products measured by LC-MS/MS ranged from 0–100 μM. (TIF) [file pone.0269572.s006.tif]

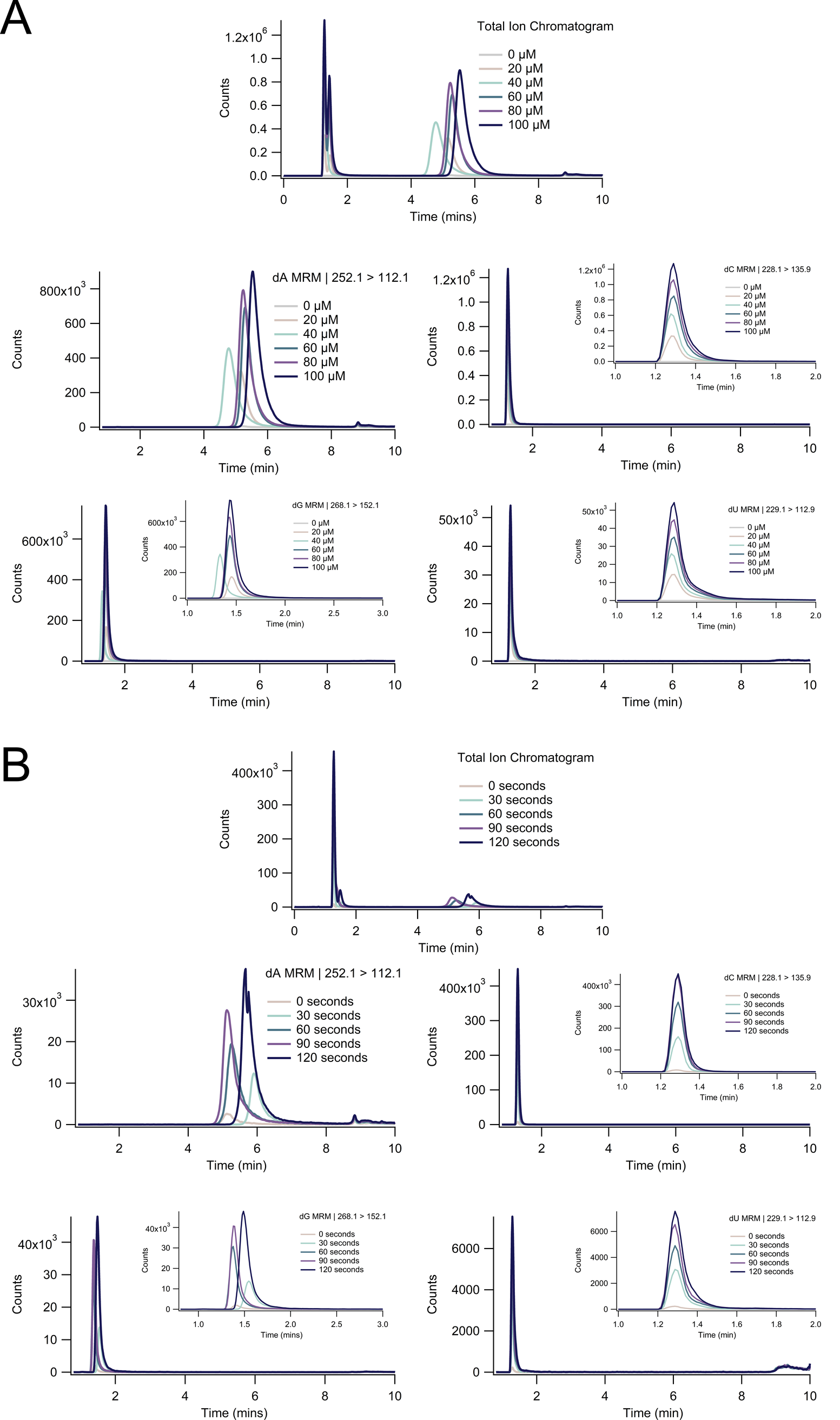

Supplement: S4 Fig — (A) TIC and MRM curves for each of the four standard curves. (B) TIC and MRM curves for the activity assay. The above condition contained Trx (30 μM), TrxR (0.5 μM), NADPH (200 μM), ATP (3000 μM), dATP (500 μM), TTP (250 μM), dGTP (100 μM), CDP (70 μM), UDP (50 μM), GDP (200 μM), ADP (110 μM), NrdA (0.1 μM dimer), and NrdB (0.5 μM dimer). (TIF) [file pone.0269572.s007.tif]

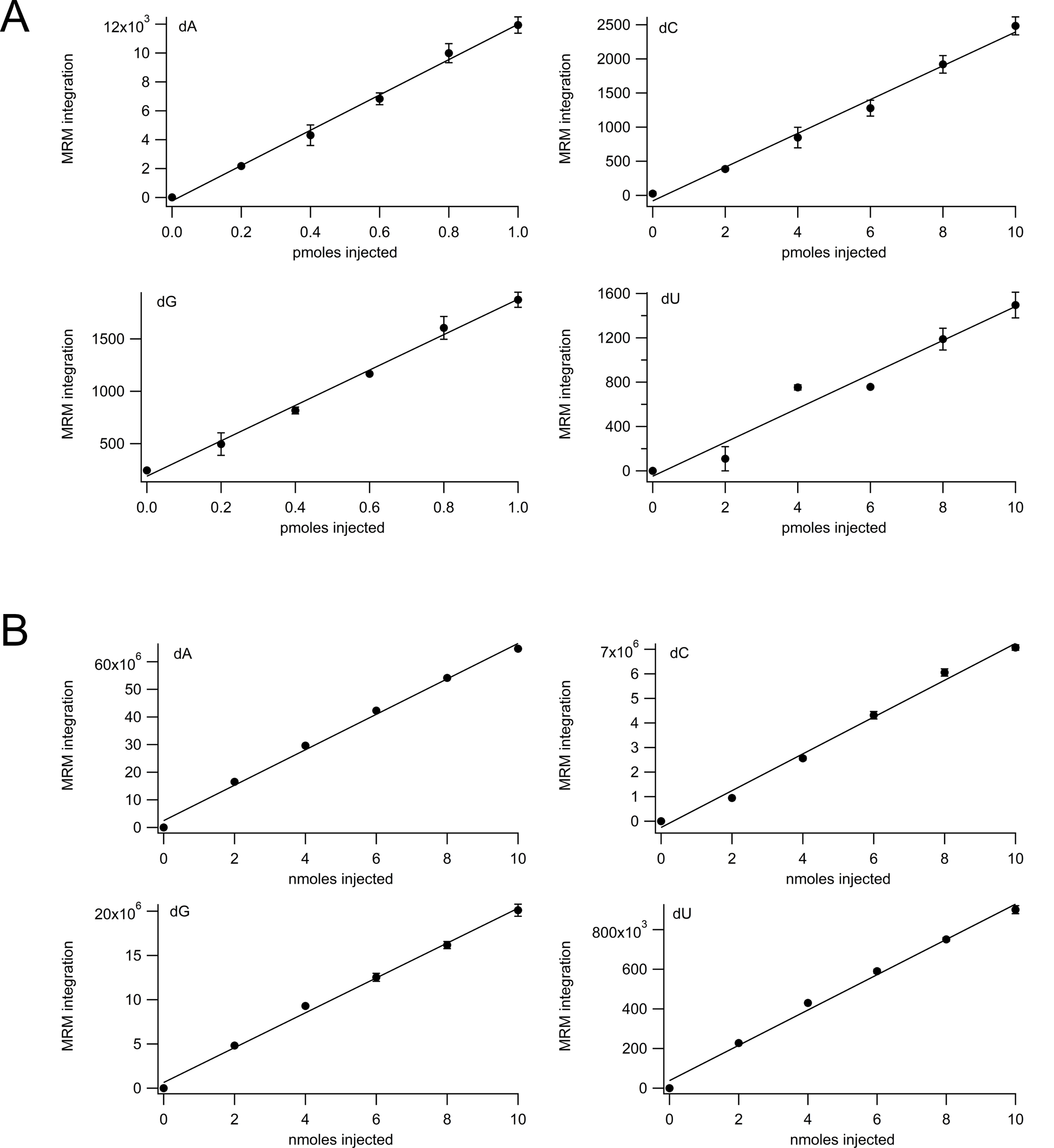

Supplement: S5 Fig — (A) Lower limit of detection for each of the four deoxynucleosides. (B) Standard curves are linear at up to 10 nmoles injected deoxyribonucleoside for each product. Note that the upper limit of detection and the upper linear range are both likely higher but are not anticipated to be relevant to RNR assays. Each data point is the mean ± standard error of the mean of three replicates. (TIF) [file pone.0269572.s008.tif]

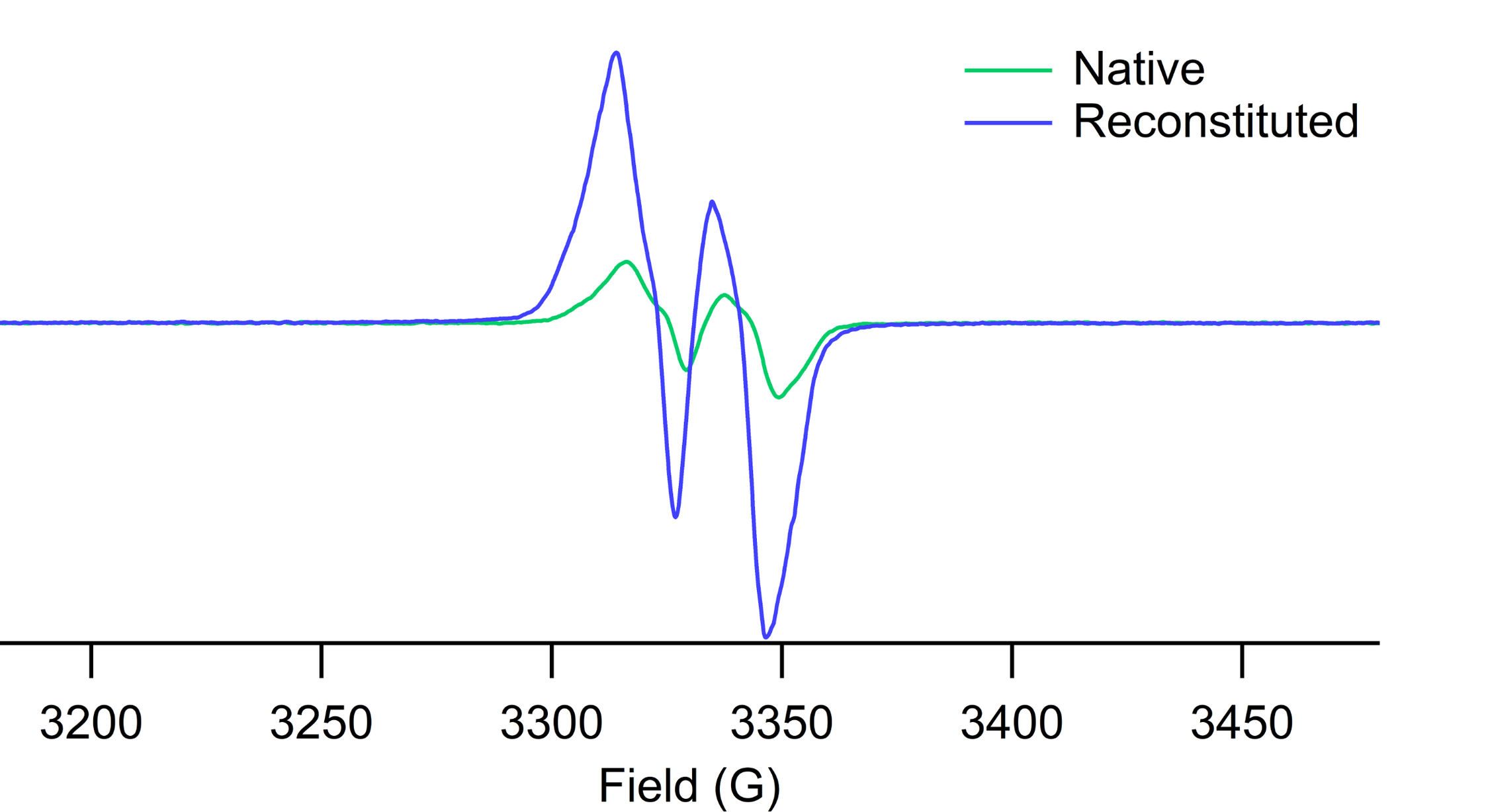

Supplement: S6 Fig — EPR spectra of native (green) and reconstituted (blue) NrdB. EPR parameters were 80K, 10 scans, center field 3330 G, sweep width 300 G, sweep time 20 s, microwave power 38dB, modulation amplitude 1.5G. Double integrals were 134.5 and 580.6 for 100 μM native and reconstituted protein, respectively, leading to radical content of 0.3 and 1.2 radicals per dimer, respectively. (TIF) [file pone.0269572.s009.tif]

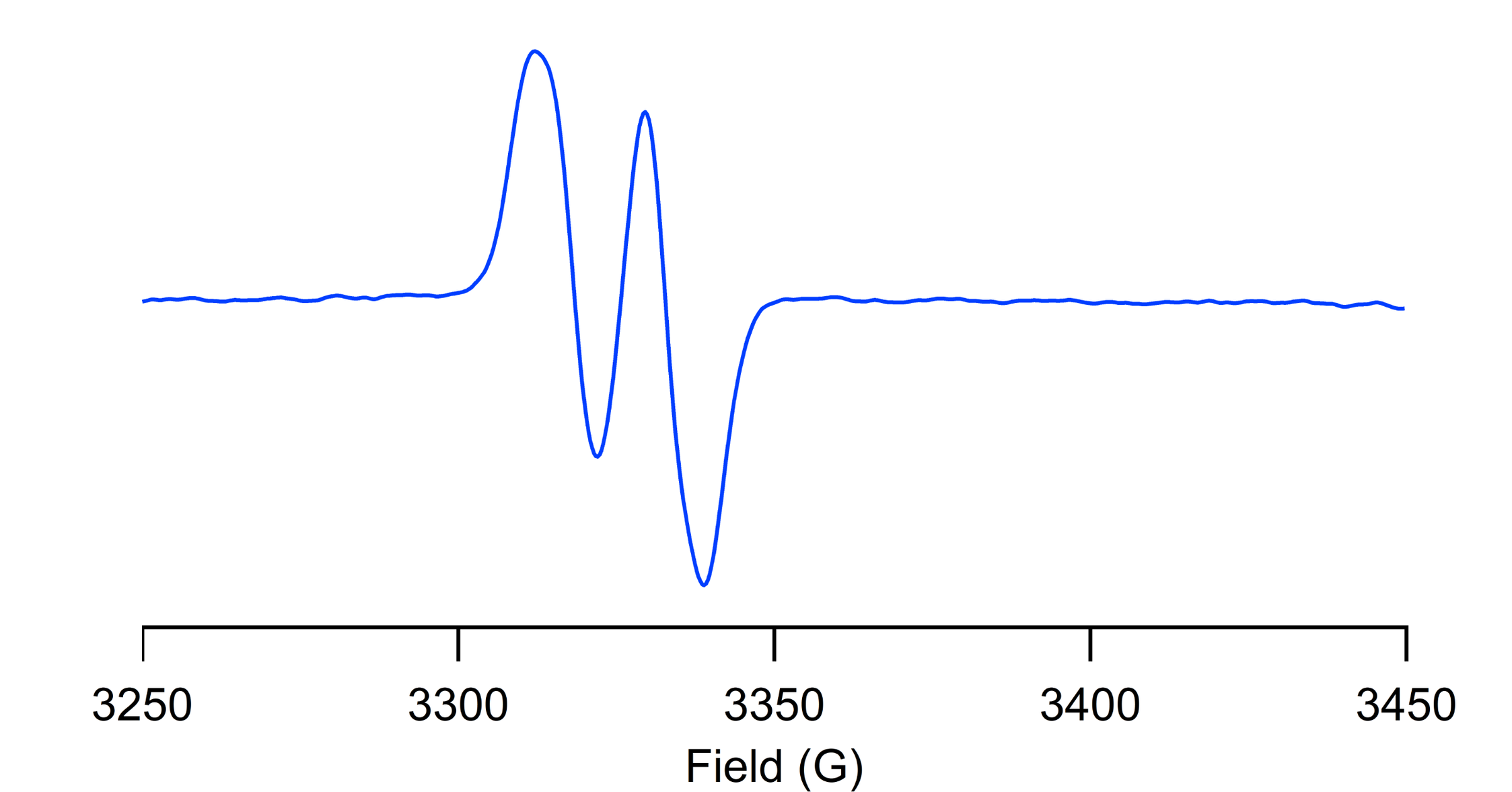

Supplement: S7 Fig — EPR parameters were 80K, 10 scans, modulation amplitude 3G, center field 3350 G, sweep width 200G, sweep time 21 s, microwave power 52dB. (TIF) [file pone.0269572.s010.tif]
