# Supplementary material for: A rapid and sensitive assay for quantifying the activity of both aerobic and anaerobic ribonucleotide reductases acting upon any or all substrates
Source: PLoS One. 2022 Jun 8;17(6):e0269572. doi: 10.1371/journal.pone.0269572 (PMC9176816; doi:10.1371/journal.pone.0269572)
Supplement: Attachment — Submitted filename: PLOS_ONE_Drennan_rev.pdf [file pone.0269572.s011.pdf]

Summary: Drennan et al. present a versatile assay for RNR activity. This assay has several benefits: it does not require radiolabeled nucleotide substrates and the associated infrastructure for working with and quantitating radioactive samples, it enables rapid and semi-high throughput product quantitation which in turn allows a wide variety of conditions to be screened, and it is applicable to a variety of RNRs from different classes and organisms. The conclusions are well-supported and are of broad interest to the readership of PLOS ONE. Suggestions to improve the clarity of the presented work are provided below, but with these emendations, I strongly support publication. The initial assay results reported already reveal important new insights into RNR allostery.

Suggested Improvements: I struggled to understand some of the basic assay details presented in the beginning of the Results section. As a 'methods' paper, I feel that these details should be explained more thoroughly in the main text. The widespread adoption of the presented assay will be enabled by an extremely clear and thorough description here. Suggestions for improvement are as follows:

- The second paragraph of the results section should be expanded to walk the reader through the analysis presented in Figure 3. Figure 3 is the first presentation of results but I found the caption difficult to correlate with the figure components. I suggest discussing it in detail in the main text and calling out the readers' attention to its various components while explaining the analysis procedure. A few questions I had were:
    - What are the different peaks in the chromatograms (at ~1.4 min and ~3.5 min)?
    - Why do retention times differ slightly for the same product?
    - The abbreviation TIC is never defined, how does it differ from MRM analysis and how is each of these detection modes used in the assay?
    - Finally, the colors of some of the traces are difficult to see. Making them thicker and darker would be very helpful.
- In the first paragraph of the results section, it should be explained that the concentrations of each reagent in the assay mixture matches those previously optimized/employed in reported radioactivity and spectrophotometric assays, and these concentrations should be provided in the main text. While normally this information is sequestered to figure captions, here, the information is of central relevance. You may even want to provide the volumes of each component (i.e. mastermix and NrdB) and the mechanism of mixing (i.e. tubes were inverted to ensure complete mixing).
- "Internal Standard" section: specify which heavy nucleotides were used. The choice of what to use will depend on the sensitivity of your LC-MS/MS instrument but it would be helpful to know what was used here and where the material was purchased (I did not see this information in the Materials section or SI).

Additional Minor Suggestions:

- Introduction section, paragraph 4, 1st sentence: in listing complicating factors phosphorylation sites, activity regulation, and oxygen sensitivity are mentioned; I would add "complex allosteric specificity relationships" to the list. The results presented here highlight this complexity!
- Introduction section, paragraph 5: In discussing the drawbacks of the spectrophotometric assay, large variability between replicates (low precision) could also be mentioned.
- Discussion section, line 253: awkward sentence. You might change to "…is more flexible in the substrates and reducing systems, and thus increases the classes of RNRs that can be assayed relative to the radioactive or coupled assays (Table 3)."
