# Supplementary material for: A rapid and sensitive assay for quantifying the activity of both aerobic and anaerobic ribonucleotide reductases acting upon any or all substrates
Source: PLoS One. 2022 Jun 8;17(6):e0269572. doi: 10.1371/journal.pone.0269572 (PMC9176816; doi:10.1371/journal.pone.0269572)
Supplement: Attachment — Submitted filename: Reviews_compiled_withresponses.pdf [file pone.0269572.s013.pdf]

Overall Response

We thank the three reviewers for their time and efforts in reviewing our manuscript. We believe that the suggestions made have improved the clarity and scope of the manuscript and have made it so that readers can more easily follow the methods presented. We have addressed each reviewer comment point-by-point below and have provided indications of changes to the manuscript using highlighting.

Reviewer #1

Summary: *Drennan et al. present a versatile assay for RNR activity. This assay has several benefits: it does not require radiolabeled nucleotide substrates and the associated infrastructure for working with and quantitating radioactive samples, it enables rapid and semi-high throughput product quantitation which in turn allows a wide variety of conditions to be screened, and it is applicable to a variety of RNRs from different classes and organisms. The conclusions are well-supported and are of broad interest to the readership of PLOS ONE. Suggestions to improve the clarity of the presented work are provided below, but with these emendations, I strongly support publication. The initial assay results reported already reveal important new insights into RNR allostery.*

We thank the reviewer for their enthusiasm. Please see below for responses to your suggestions for improvement.

Suggested Improvements: *I struggled to understand some of the basic assay details presented in the beginning of the Results section. As a 'methods' paper, I feel that these details should be explained more thoroughly in the main text. The widespread adoption of the presented assay will be enabled by an extremely clear and thorough description here.*

Suggestions for improvement are as follows:

- *The second paragraph of the results section should be expanded to walk the reader through the analysis presented in Figure 3. Figure 3 is the first presentation of results but I found the caption difficult to correlate with the figure components. I suggest discussing it in detail in the main text and calling out the readers' attention to its various components while explaining the analysis procedure. A few questions I had were:*
    - *What are the different peaks in the chromatograms (at ~1.4 min and ~3.5 min)?*
      We have added in a reference in the text to S2 Table, which lists the retention time for each product. We have also added a sentence in the figure legend to Figure 3 stating that the peak at 1.4 min is dC and the peak at 3.5 min is A.
    - *Why do retention times differ slightly for the same product?*
      We see slight variability in retention times as indicated in S2 Table for some products; however, retention time is not the primary form of product identification (due to the precision of MRM mode in isolating product signal) and so this variability is tolerable in the assay and we have not investigated the source of this variability. We have added in a sentence in the text explaining this.
    - *The abbreviation TIC is never defined, how does it differ from MRM analysis and how is each of these detection modes used in the assay?*
      We have now defined TIC (total ion chromatogram) both in the text and figure legend and added a paragraph on MRM analysis and extraction of MRM curves from TICs.
    - *Finally, the colors of some of the traces are difficult to see. Making them thicker and darker would be very helpful.*

We have made the traces thicker in Figures 3, 5, 6, 7, and S3 and S4, and have changed the color of the lightest trace(s) in Figures 3, 8, S3, and S4 to increase visibility for readers.

- *In the first paragraph of the results section, it should be explained that the concentrations of each reagent in the assay mixture matches those previously optimized/employed in reported radioactivity and spectrophotometric assays, and these concentrations should be provided in the main text. While normally this information is sequestered to figure captions, here, the information is of central relevance. You may even want to provide the volumes of each component (i.e. mastermix and NrdB) and the mechanism of mixing (i.e. tubes were inverted to ensure complete mixing).*

  For new RNRs, there may be a period of optimization for users to determine what conditions generate a linear activity curve within the bounds of the standard curve used and so exact concentrations may need to be varied. We have added the phrase "under the same nucleotide conditions" in the *"E. coli* class Ia RNR activity assay results…" section to highlight that the nucleotide conditions are the same as optimized assays since other sections (i.e. in looking at physiological concentrations and measuring *St*NrdD activity) use other nucleotide concentrations that were not the same as those optimized in the spectrophotometric and radioactive assays.

  We have added in the mixing strategy and mastermix and initiator volumes and have added in concentrations of assay components in parentheticals to the first paragraph.

- *"Internal Standard" section: specify which heavy nucleotides were used. The choice of what to use will depend on the sensitivity of your LC-MS/MS instrument but it would be helpful to know what was used here and where the material was purchased (I did not see this information in the Materials section or SI).*

  We have added that the heavy nucleotides used were $^{13}C/^{15}N$ in the main text and in the legend for Figure 6. This information, along with where the material was purchased (Santa Cruz Biotechnology), was already present in the materials and methods.

Additional Minor Suggestions:

- *Introduction section, paragraph 4, 1st sentence: in listing complicating factors phosphorylation sites, activity regulation, and oxygen sensitivity are mentioned; I would add "complex allosteric specificity relationships" to the list. The results presented here highlight this complexity!*

  We have added this to the list of complicating factors.

- *Introduction section, paragraph 5: In discussing the drawbacks of the spectrophotometric assay, large variability between replicates (low precision) could also be mentioned.*

  We have added this as a drawback to using the coupled assay.

- *Discussion section, line 253: awkward sentence. You might change to "…is more flexible in the substrates and reducing systems, and thus increases the classes of RNRs that can be assayed relative to the radioactive or coupled assays (Table 3)."*

  We have modified the sentence as suggested.

Reviewer #2:

The presented manuscript describes the development of an LC-MS/MS activity assay for the partial characterisation of ribonucleotide reductases (RNs) by making use of their intrinsic allosteric inhibition which determines the product distribution. Thus, this method can assay RNs conversion of ribonucleotides (substrates) in the presence of all four at the same time. Further, the method is faster and does not require handling of radioactive materials. Overall, the manuscript is very well written and interesting to read. I am not an expert in the field but had overall no difficulties understanding it. My only suggestion is a linguistic one – the meaning in line 347, I had to read a couple of times until I got the pieces together. Would it be possible changing "prior to use of heat" to "before using/applying heat"?

We have changed the wording to "before using heat".

Recommend for publication as it stands.

Reviewer #3:

The motivation for this methodological paper is that RNR activity assays in use are either using radioactivity and/or can only assay one substrate at a time. As an additional advantage of the assay presented here the authors stress in the title of the paper that their assay can be used for both aerobic and anaerobic ribonucleotide reductases. Simple and multiple purpose assays are beyond doubt welcome within the RNR field. However, several claims in this paper are not true, and it is not unique with an assay that can be used for aerobic and anaerobic RNRs alike. Many research groups within the RNR field have reported using an HPLC assay (Hofer 1998, Rofougaran 2008, Crona 2016, Loderer 2017 & 2019, Srinivas 2018, Rozman Grinberg 2018x2, 2019, Martinez-Carranza 2020, Rehling 2022), which the authors of this manuscript refrain from acknowledging until lines 295-301, where an older version of the HPLC assay is referred to in negative terms. The HPLC assay has been used for multiple substrate assays and for anaerobic assays. It seems the "canonical assays" referred to in the current paper mostly refer to what has been used in the authors' research groups and by the authors' close collaborators.

As detailed in the point-by-point below, we have expanded the introduction and the discussion of this manuscript to include additional information about the previously employed HPLC assays.

Major comments:

1) *Abstract: There are multiple substrate assays that have been published and are in use by other research groups. Is the radioactive assay commonly used nowadays?*

Yes. The radioactive assay is still used, but we have edited the abstract to make it clear that we are comparing this assay to a number of published assays including the radioactive assay.

2) The LC-MS/MS assay is claimed to be cost effective, but for many of the exemplified applications the authors are using heavy nucleotides as internal standards, which conceivably increase the costs.

We have calculated the amount that heavy nucleotides cost per reaction both at our time of purchase and at the current time if a lab were going to purchase them (all from Santa Cruz Biotech, our supplier noted in the methods). The addition of a single heavy nucleotide as an internal standard adds approximately 25 – 50 cents per triplicate 5-timepoint assay, which is likely not an increase in cost that is unmanageable for researchers with access to the other tools and reagents necessary for RNR assays.

Heavy nucleotide	price ($) [SCBT, 6/16/2020]	molecular weight	concentration (mM)	mg	volume (uL)	volume at 1.6 mM (uL)	Price per 1 uL addition (cents)	Price per 5-timepoint assay (cents)	Price per 5-timepoint assay in triplicate (cents)			
dA	257	506.8	100	10	197.32	12332.28	2.1	10	31			
T	285	494.08	100	10	202.40	12649.77	2.3	11	34			
dG	209	522.07	100	10	191.55	11971.57	1.7	9	26			
							6.1	30.4	91.2	<sum for addition of all		

Heavy nucleotide	price ($) [SCBT, 3/24/2022]	molecular weight	concentration (mM)	mg	volume (uL)	volume at 1.6 mM (uL)	Price per 1 uL addition (cents)	Price per 5-timepoint assay (cents)	Price per 5-timepoint assay in triplicate (cents)			
dA	345	506.8	100	10	197.32	12332.28	2.8	14	42			
T	368	494.08	100	10	202.40	12649.77	2.9	15	44			
dG	360	522.07	100	10	191.55	11971.57	3.0	15	45			
							8.7	43.6	130.7	<sum for addition of all		

3) *A general impression that any reader would get from this paper is that only the authors and the authors' close colleagues have measured RNR activity in later years, whereas reference to other scientists within the RNR field primarily refers to studies performed before the turn of the millennium. However, this is certainly not the case, as exemplified by papers listed above of authors using the HPLC assay to measure RNR enzyme activity.*

We have added a paragraph in the introduction addressing these concerns and additionally highlighting the assays that already exist in the field and the contributions they have made. We did not include Hofer 1998 or Crona 2016 in these references because both of these references use HPLC to measure NTP/dNTP pools as opposed to RNR activity; however, we have incorporated the rest of the references supplied into the text and moved some of our other references to HPLC-based assays that were already present to the introduction. We strove to acknowledge both the contributions to the field that these assays have made as well as the limitations that led us to spend a number of years developing the presented assay.

4) *Stopping the assay by temperature inactivation may not work well for thermostable RNRs. The authors have chosen an RNR that is inactivated at 50°C, but do not discuss properly how to deal with thermostable RNRs in general. The authors cannot measure the time to reach their stopping temperature with the current set up (footnote to Table S1), and simply skip over that problem. Is it not possible that proteins refold and nucleotide reduction can resume after the short incubation at 95°C?*

Clearly, we agree with the reviewer that heat inactivation can be problematic for thermostable RNRs. It was for this reason that we provided an SI Table 1 and SI figure 1 and discussed this issue in the text. Given that this reviewer wants additional discussion, we have added a sentence into the discussion to provide readers with other options for stopping the reaction and other control experiments that can be performed:

> Alternatively, heating-independent methods for stopping the reaction can be employed such as acid, methanol, or cold [11,21,44,58,59,61,63]. To verify that the method used to

stop the reaction is satisfactory and that no refolding of the enzyme has occurred, the enzyme can be assayed following "inactivation" to ensure that there is no measured activity.

In terms of the "stopping temperature," although the temperature of the PCR machine is set to 95 °C, the "stopping temperature" of the reaction is the temperature at which the RNR is inactivated. We noted previously in the discussion that even if there is a slight delay in inactivation (because of the time it may take to reach the desired temperature) that the slope of the line generated will be consistent and thus activity can still be accurately measured. We were able to successfully measure the time it took to reach both 60 °C, which is 10 °C higher than the melting temperature (and thus "stopping temperature") of the thermostable protein used in the experiments herein, and 85 °C, which is 35 °C higher than the aforementioned melting temperature; thus, although the PCR machine is set to 95 °C, we are less concerned about the time it takes to reach 95 °C than the time it takes to reach a temperature where the protein(s) is(/are) inactivated. This discrepancy in "block set temperature" and true "stopping temperature" is why we did not further pursue the exact time it took for the solution to reach 95 °C. We added a sentence explaining this to footnote b in S1 Table.

5) *The authors claim using physiological nucleotide concentrations in several of their experiments, but never specify what the physiological nucleotide concentrations in Escherichia coli or Streptococcus thermophilus are. The paper must be updated with physiological nucleotide concentrations.*

All physiological nucleotide concentrations for *E. coli* were modeled after Buckstein (2008) and Traut (1994), which are referenced throughout the text, and the exact concentrations in each experiment are indicated in the figure legends and text. However, to make the previously-measured concentrations even clearer, we added sentences in the results immediately after the first time that we discuss approximately physiological concentrations of nucleotides to call out the studies by name rather than just having the references.

Since there is no one correct number for physiological concentrations, we added the word "approximately" before physiological in all relevant spots in the text.

We do not make any claims about the physiological concentrations of nucleotides in *S. thermophilis*, as that has not been measured to our knowledge.

6) *Lines 59-60: The authors mention that most class II RNRs use NDP substrates; this refers primarily to which class II RNRs have been studied so far. Schell & al 2021 published a method to predict whether a class II RNR uses NDPs or NTPs for substrate and could even suggest the way these differences have evolved. The statement on lines 59-60 needs to be backed up with proper reference(s).*

We reference the work done characterizing class II RNRs as is appropriate and proper. Based on this reviewer's suggestion, we have also added a reference to the 2021 prediction paper. Perhaps this assay will help authors of that work valid their predications.

7) *Line 63: The authors mention the physiological thioredoxin system, but forget the glutaredoxin system, which they list incorrectly in Table 1. In Table 1 they list glutaredoxin as if it would be reduced by thioredoxin reductase, which is not correct.*

We have moved "glutaredoxin" in Table 1 to avoid confusion.

8) *Lines 92-100: The authors mention that one drawback with the boronate assay is that it can only be used for CDP, which is incorrect.*

We were talking about a drawback of the radioactive assay with respect to CDP. We were not talking about the boronate step that separates out substrates from products. We have changed the sentence to read "this *radioactive* assay" to clarify this point.

9) *Lines 149-154: It is impossible to understand what figure S3 shows from the description here and from the figure legend.*

Figure S3 shows the standard curve optimization. We have changed the title of figure to "standard curve optimization." Before we gave the bottom line of what was important to optimize. That information is now sentence 2.

10) Line 162, 265-267: The obtained specific activity of EcNrdB (3600 nmol/min x mg) is 30-55% lower than reported in "established assays". This is especially confusing as the authors claim that their protein contains 1.2 radical per dimer, so ought to be fully active (or is the maximal radical content assumed to be 2 radicals per dimer?). It becomes even more confusing in lines 193-194 (and lines 965-967), when the same NrdB preparation suddenly has a specific activity of 8000-9000 nmol/min x mg), The authors need to explain these remarkable discrepancies.

We have repeated the assay under Figure 4 conditions many times and the low value of 3600 nmol / min x mg with NrdB limiting is an anomaly. We got this lower value early on and didn't update the figure. The reviewer is correct that it is confusing to show these original data. We have now updated figure 4 to show more the typical activity values: 10000 ± 1000 nmol / min x mg and 10100 ± 700 nmol / min x mg, which are closer to the values in the experiment shown in Figure 6 (8300 ± 700 nmol / min x mg and 8900 ± 700 nmol / min x mg). Since these four independent results were all within a standard deviation of one another, the 3600 nmol / min x mg result does seem to be an anomaly, perhaps due to poor handling of beta during this experiment. We recognize that these values are slightly higher than the literature values, potentially due to higher iron or radical installation in these proteins. Note: we believe that NrdB must have 2 radicals/dimer to be active; NrdB either has 0 radicals or 2 radicals. 1.2 radicals/dimer means that there is only slightly more active NrdB (2 radicals) in the mix than inactive NrdB (0 radical).

11) *Lines 167-168: Why do comparison to results obtained with other assays only refer to the authors' own studies which show relatively high values, when there are numerous other studies that report much lower activity values for dATP inhibited RNR.*

We had a difficult time finding the studies that are referred to in the above comment. There are multiple studies on different RNRs (either class Ia RNRs not from *E. coli* or different classes of *E. coli* RNRs), but we could not find studies on *E. coli* NrdA that gave values for dATP inhibition at similar concentrations as used in this paper. We did find two studies that graphed relative activity over a range of dATP concentrations and took those data points closest to 175 µM (which appeared to be at 100 µM) to compare the relative activity; we added a sentence in the results referencing those papers (Rofougaran et al 2008 and Birgander et al 2004). The comparisons to our own studies are the closest comparisons due to the greatest similarities in protein expression and purification strategies; however, we would be happy to compare to relevant numbers found by other groups should they be provided.

12) Lines 172-177: How do the activity values for the class III RNR used here compared to specific activities for other class III RNRs?

We have added in comparisons to activities of other class III RNRs and added in a comparison sentence to the discussion.

13) *Lines 204-223: Here the reader is faced with a multitude of results of variations of nucleotide concentrations, but completely left without any explanation for what might be the reason for these results. The discussion also lacks hypotheses on this subject.*

We have added notes on comparisons to physiological concentrations (see response to point below) to allow the reader to draw their own conclusions based on the results section. We present our interpretations of these results and the results of all the 4 dN assays, which closely match expected outcomes based on past research on substrate/effector pairs, in the discussion section (three paragraphs, the first starting with "using the ability to measure…"); we have added some notes on physiological relevance (see response to point below) to the discussion in order to make clearer the conclusions that can be drawn from looking at activity and/or specificity effectors at different concentrations under an otherwise close-to-physiological nucleotide milieu.

We focused in the discussion on how the results obtained match previously known conclusions about the enzyme, which they overwhelmingly do, and did not go deeper into hypotheses on individual nucleotide concentrations in order to keep the focus of the paper on method development and validation.

14) *Lines 233-243: Here the reader is presented with results that positive allosteric effects levels off at increasing effector nucleotide concentrations but is left without hypotheses or explanations. This is even more confusing as physiological levels of nucleotides have not been mentioned*

anywhere in the paper, so it is not possible to judge if such high effector concentrations are physiologically meaningful or not.

We have added values from the literature for the physiological levels of nucleotides (see response to comment 5). When we looked at broad nucleotide concentration ranges, we did go above the physiological range. Instead of saying "high" levels of given nucleotide, we now say "supraphysiological," so that it will be clear to readers that these concentrations are higher than the physiological range.

15) *Lines 250-251: This statement is not true (see numerous examples listed above).*

We believe that all the people who traveled to JoAnne Stubbe's lab from all over the world to learn how to assay RNR would say that the "field has been challenged by the lack of an accessible assay." We have had numerous labs contact us to assay RNR against a compound that they think might be inhibiting that RNR because they couldn't get an assay to work. Thus, we respectfully disagree that the statement that the "field has been challenged" is untrue. However, we have adjusted the sentence in question to say "…lack of an accessible <u>and validated</u> assay" to emphasize the validation against the established *E. coli* activity assay values that have not been completed with other HPLC-based assays. We also added a sentence at the end of the paragraph specifically addressing the ways that the presented assay improves on HPLC-based assays.

16*) Lines 254-256: What is meant by "various ratios …, different substrates/effectors…., different temperatures…. can be assayed in A SINGLE EXPERIMENT*"?

We have removed the "in a single experiment" from the sentence since it was confusing.

17) *Lines 339-342: It is stated that the inclusion of heavy nucleotides cannot be used when performing multiple substrate assays, so it appears that other assays might be superior in these cases.*

This assay is still advantageous when same-day standard curves are used instead of heavy nucleotides. We have added sentences, saying that standard curves can be generated in advance and in (relative) bulk and stably stored so that it is not a large burden for the researcher to run them prior to any samples on the LC-MS/MS.

Other HPLC assays that are in the literature also need standard curves to correlate signal to amount of product generated. The time necessary to run the standard curves using the presented LC-MS/MS assay (10 minutes per run, so ~3 hours per triplicate standard curve) is greatly surpassed by the extra time it takes to run each sample on the HPLC for HPLC-based assays (180 minutes per run for Rehling et al 2022; 72 minutes per run for Rofougaran et al 2008; 45 minutes per run for Hendricks and Mathews 1997), making the LC-MS/MS assay data generation still faster (and less resource-intensive in terms of buffer components and potentially instrumentation costs) than previous HPLC assays that do not require same-day standard curves.

18) *Lines 653-656: How is the specific activity of Streptococcus thermophilus adjusted for glycyl radical content, when the glycyl radical content is claimed to be 1.5 radical per dimer (line 951) which to this referee is the highest radical content ever achieved in any RNR. Is the maximal glycyl radical content assumed to be 1 radical per dimer or 2 radicals per dimer?*

We adjusted to the theoretical maximum of 2 radicals per dimer. We have indicated this in the sentence:

> … with total mg NrdD adjusted based on concentration of glycyl radical identified in EPR analysis *assuming maximal radical concentration of 2 radicals per dimer*, in each 10 µL injection to yield nmoles/mg-min for each time course …

The higher glycyl radical content is outside the scope of this paper, but we are planning on exploring this further in future class III RNR-focused publications. The only report in the literature of glycyl radical content in a class III RNR is for the *L. lactis* RNR in Torrents et al. 2001, where 0.5 glycyl radicals per monomer (1 per dimer) were reported. We do note that although we generally do not see higher than 1.2 radicals per dimer in reconstituted *E. coli* NrdB*,* the active structure of *E. coli* suggests that two radicals per dimer are necessary for turnover on both alpha subunits, and so the theoretical maximum radical concentration still remains 2 radicals per dimer. The class III RNR may have a similar or different mechanism, which remains to be discovered, but we used the theoretical maximum of 2 radicals per dimer due to a lack of conclusive evidence against this maximum.

19) Table 3: *The authors need to qualify the expression "canonical RNR activity assays".*

We have removed the word canonical and have replaced it with "Comparison of the *radioactivity and coupled* RNR activity assays…"

20) *Figure 1: The references in brackets [1-4] seem to be referring to references listed in the Supplement and not in the Reference list. This must be corrected.*

The references in the figure legends didn't get updated when we moved the figures to the main text before submission. We apologize. We have now updated the references in the figure legends and removed reference from the supplement.

Minor points:

1) *Lines 180-181, 224-226, 611-613: Incomplete sentences*

We have rewritten these three sentences.

2) *Line 362: specify which stoichiometries are meant*

We have rearranged the sentence so that it is clearer:

> Future applications of LC-MS/MS for the study of RNRs include *determinations of the stoichiometries of nucleotides binding to RNRs and their associated KDS*.

3) *Last sentence of the Discussion can be deleted.*

We have chosen to keep this sentence.

4) *Lines 572-573: What is the logic for increasing Trx concentration to compensate for His tags?*

We rephrased this sentence to hopefully be clearer:

> All proteins used in these assays have intact His tags, but it has been shown that His tags do not substantially interfere with NrdA and NrdB activity [72]. To ensure that re-reduction was not the rate-limiting step, we used a large excess (60x) of Trx to ensure full and rapid reduction of NrdA.

5) *Line 603: Has StNrdD been separated from StNrdG?*

The StNrdD was not separated from the StNrdG prior to use in anaerobic assays as is the common practice. We added a note regarding this in the anaerobic assay method section:

> In an anaerobic chamber (Mbraun) protein mastermix containing buffer, DTT (5 mM), sodium formate (12.5 mM), and activated StNrdD (0.10 µM; *still in mixture with StNrdG from the activation reaction*) and a nucleotide mastermix containing all nucleotides needed…

6) *Line 894: Metallocofactors are not specified in Table 1*.

We have removed the part of the sentence in the figure legend that indicates that metallocofactors were specified in Table 1.

7) *Figure 5A: How many replicates were used.*

We added that there were three replicates used per data point.

8) Figure 7C: dU bar cannot be seen in figure.

We meant to show the bar graph in 7C that went with data in 7B, but we didn't. We apologize for this mistake and thank the reviewer for bringing our attention to Panel 7C. We have corrected the 7C graph and the dU bar is now visible.
