# Supplementary material for: A rapid and sensitive assay for quantifying the activity of both aerobic and anaerobic ribonucleotide reductases acting upon any or all substrates
Source: PLoS One. 2022 Jun 8;17(6):e0269572. doi: 10.1371/journal.pone.0269572 (PMC9176816; doi:10.1371/journal.pone.0269572)
Supplement: Attachment — Submitted filename: Response to reviewers 2.docx [file pone.0269572.s014.docx]

Response to one remaining reviewer comment

Reviewer #3: The authors have responded satisfactorily to all questions, except for one point. It is still not clear from the determination of protein activity from LC-MS/MS data how the activity of E. coli NrdB is calculated. The radical content of EcNrdB was determined to 1.2 radicals per dimer, and the authors state in their response that “we believe that NrdB must have 2 radicals/dimer to be active”. Is the activity of 10 000 nmol dCDP/mg-min for EcNrdB adjusted for radical content or not? The question is valid, as the activity of S. thermophilus NrdD is adjusted for radical content. Whether the activity of EcNrdB has been adjusted for radical content or not needs to be clearly spelled out in the section on lines 252-266 where EcNrdB activity is reported or in the section on lines 855-864 where the adjustment of StNrdD activity is described.

The activity of 10 000 nmol dCDP / mg-min for *Ec*NrdB is not adjusted for radical content. We had added a sentence describing this in the same area of the methods where the adjustment is described for NrdD:

The slope was divided by the total mg of *the limiting protein present* in each 10 µL injection to yield nmoles/mg-min for each time course. *For aerobic assays, limiting protein was total mg of either NrdA or NrdB (not adjusted for radical content); for anaerobic assays, limiting protein was* total mg activated NrdD present, with total mg NrdD adjusted based on concentration of glycyl radical identified in EPR analysis assuming maximal radical concentration of 2 radicals per dimer.
