# Supplementary material for: A rapid and sensitive assay for quantifying the activity of both aerobic and anaerobic ribonucleotide reductases acting upon any or all substrates
Source: PLoS One. 2022 Jun 8;17(6):e0269572. doi: 10.1371/journal.pone.0269572 (PMC9176816; doi:10.1371/journal.pone.0269572)
Supplement: Attachment — Submitted filename: Reviewer comments PONE-D-22-03785.pdf [file pone.0269572.s012.pdf]

Reviewer comments PONE-D-22-03785:

The motivation for this methodological paper is that RNR activity assays in use are either using radioactivity and/or can only assay one substrate at a time. As an additional advantage of the assay presented here the authors stress in the title of the paper that their assay can be used for both aerobic and anaerobic ribonucleotide reductases. Simple and multiple purpose assays are beyond doubt welcome within the RNR field. However, several claims in this paper are not true, and it is not unique with an assay that can be used for aerobic and anaerobic RNRs alike. Many research groups within the RNR field have reported using an HPLC assay (Hofer 1998, Rofougaran 2008, Crona 2016, Loderer 2017 & 2019, Srinivas 2018, Rozman Grinberg 2018x2, 2019, Martinez-Carranza 2020, Rehling 2022), which the authors of this manuscript refrain from acknowledging until lines 295-301, where an older version of the HPLC assay is referred to in negative terms. The HPLC assay has been used for multiple substrate assays and for anaerobic assays. It seems the "canonical assays" referred to in the current paper mostly refer to what has been used in the authors' research groups and by the authors' close collaborators.

Major comments:

1) Abstract: There are multiple substrate assays that have been published and are in use by other research groups. Is the radioactive assay commonly used nowadays?
2) The LC-MS/MS assay is claimed to be cost effective, but for many of the exemplified applications the authors are using heavy nucleotides as internal standards, which conceivably increase the costs.
3) A general impression that any reader would get from this paper is that only the authors and the authors' close colleagues have measured RNR activity in later years, whereas reference to other scientists within the RNR field primarily refers to studies performed before the turn of the millennium. However, this is certainly not the case, as exemplified by papers listed above of authors using the HPLC assay to measure RNR enzyme activity.
4) Stopping the assay by temperature inactivation may not work well for thermostable RNRs. The authors have chosen an RNR that is inactivated at 50°C, but do not discuss properly how to deal with thermostable RNRs in general. The authors cannot measure the time to reach their stopping temperature with the current set up (footnote to Table S1), and simply skip over that problem. Is it not possible that proteins refold and nucleotide reduction can resume after the short incubation at 95°C?
5) The authors claim using physiological nucleotide concentrations in several of their experiments, but never specify what the physiological nucleotide concentrations in Escherichia coli or Streptococcus thermophilus are. The paper must be updated with physiological nucleotide concentrations.
6) Lines 59-60: The authors mention that most class II RNRs use NDP substrates; this refers primarily to which class II RNRs have been studied so far. Schell & al 2021 published a method to predict whether a class II RNR uses NDPs or NTPs for substrate and could even suggest the way these differences have evolved. The statement on lines 59-60 needs to be backed up with proper reference(s).
7) Line 63: The authors mention the physiological thioredoxin system, but forget the glutaredoxin system, which they list incorrectly in Table 1. In Table 1 they list glutaredoxin as if it would be reduced by thioredoxin reductase, which is not correct.
8) Lines 92-100: The authors mention that one drawback with the boronate assay is that it can only be used for CDP, which is incorrect.

9) Lines 149-154: It is impossible to understand what figure S3 shows from the description here and from the figure legend.
10) Line 162, 265-267: The obtained specific activity of EcNrdB (3600 nmol/min x mg) is 30-55% lower than reported in “established assays”. This is especially confusing as the authors claim that their protein contains 1.2 radical per dimer, so ought to be fully active (or is the maximal radical content assumed to be 2 radicals per dimer?). It becomes even more confusing in lines 193-194 (and lines 965-967), when the same NrdB preparation suddenly has a specific activity of 8000-9000 nmol/min x mg), The authors need to explain these remarkable discrepancies.
11) Lines 167-168: Why do comparison to results obtained with other assays only refer to the authors’ own studies which show relatively high values, when there are numerous other studies that report much lower activity values for dATP inhibited RNR.
12) Lines 172-177: How do the activity values for the class III RNR used here compared to specific activities for other class III RNRs?
13) Lines 204-223: Here the reader is faced with a multitude of results of variations of nucleotide concentrations, but completely left without any explanation for what might be the reason for these results. The discussion also lacks hypotheses on this subject.
14) Lines 233-243: Here the reader is presented with results that positive allosteric effects levels off at increasing effector nucleotide concentrations but is left without hypotheses or explanations. This is even more confusing as physiological levels of nucleotides have not been mentioned anywhere in the paper, so it is not possible to judge if such high effector concentrations are physiologically meaningful or not.
15) Lines 250-251: This statement is not true (see numerous examples listed above).
16) Lines 254-256: What is meant by “various ratios …, different substrates/effectors…., different temperatures…. can be assayed in A SINGLE EXPERIMENT”?
17) Lines 339-342: It is stated that the inclusion of heavy nucleotides cannot be used when performing multiple substrate assays, so it appears that other assays might be superior in these cases.
18) Lines 653-656: How is the specific activity of Streptococcus thermophilus adjusted for glycyl radical content, when the glycyl radical content is claimed to be 1.5 radical per dimer (line 951) which to this referee is the highest radical content ever achieved in any RNR. Is the maximal glycyl radical content assumed to be 1 radical per dimer or 2 radicals per dimer?
19) Table 3: The authors need to qualify the expression “canonical RNR activity assays”.
20) Figure 1: The references in brackets [1-4] seem to be referring to references listed in the Supplement and not in the Reference list. This must be corrected.

Minor points:

1) Lines 180-181, 224-226, 611-613: Incomplete sentences
2) Line 362: specify which stoichiometries are meant
3) Last sentence of the Discussion can be deleted.
4) Lines 572-573: What is the logic for increasing Trx concentration to compensate for His tags?
5) Line 603: Has StNrdD been separated from StNrdG?
6) Line 894: Metallocofactors are not specified in Table 1.
7) Figure 5A: How many replicates were used.
8) Figure 7C: dU bar cannot be seen in figure.
